# Tripterygium wilfordii multiglycosides combined with prednisone in the treatment of idiopathic membranous nephropathy: A protocol for a systematic review and meta-analysis

**DOI:** 10.1097/MD.0000000000018970

**Published:** 2020-01-31

**Authors:** Yuxia Jin, Jiayuan Zhang, Yunxia Wang, Xiao Xiao, Qi Zhang

**Affiliations:** Chengdu University of Traditional Chinese Medicine, Jinniu District, Chengdu, Sichuan, China.

**Keywords:** idiopathic membranous nephropathy, protocol, systematic review, traditional Chinese medicine, tripterygium wilfordii multiglycosides

## Abstract

**Aim:**

The aim of this review is to assess the efficacy and safety of tripterygium wilfordii multiglycosides combined with prednisone in the treatment of idiopathic membranous nephropathy.

**Background:**

Tripterygium wilfordii multiglycosides, a Chinese patent medicine, is widely in-depth research in China, and is proved to have anti-inflammatory and immunosuppressive effect. It has been extensively used in China for the treatment of autoimmune diseases, such as idiopathic membranous nephropathy (IMN). However, there has no relevant systematic review studied on its effects and safety been reported. We plan to perform a systematically reviewing to assess the efficacy and safety of tripterygium wilfordii multiglycosides combined with hormones in the treatment of IMN.

**Methods:**

Seven electronic databases will be searched to identify eligible trials. Randomized controlled trials (RCTs) that compared tripterygium wilfordii multiglycosides combined with prednisone versus standard therapy are included. Methodological quality is assessed using the Cochrane Collaboration Risk of Bias tool. A random- or fixed-effect model is used to analyze outcomes that are expressed as risk ratios (RRs) or mean differences (MD), and the *I*^2^ statistic is used to assess heterogeneity.

**Results:**

A high-quality synthesis of current evidence of tripterygium wilfordii multiglycosides combined with prednisone in the treatment of idiopathic membranous nephropathy will be provided in this study.

**Conclusion:**

This systematic review will provide evidence of whether tripterygium wilfordii multiglycosides is an effective intervention for idiopathic membranous nephropathy.

PROSPERO registration number: No.CRD42018118179.

## 1 Introduction

Idiopathic membranous nephropathy (IMN) is one of the most common causes of nephrotic syndrome. Although 30% of IMN patients can complete remission (CR) or partial remission (PR) spontaneously,^[1,2]^ there are still 30% to 40% of them with continuous urinary protein, and can progress to end-stage renal disease (ESRD).^[3]^ According to the KDIGO (Kidney Disease: Improving Global Outcomes), the nephrotic syndrome of IMN patients can be relieved by the combination of hormones and cytotoxic drugs (chlorambucil or oral cyclophosphamide [CTX]).^[4]^ However, possible side effects of this standard treatment, including infection, thrombosis, increased risk of cancer, and myelosuppression can cause patients to refuse treatment. 67% of the patients had at least 1 adverse drug reactions (ADR) after CTX treatment and 10% discontinued treatment for severe ADR.^[5–7]^ Although a variety of studies have demonstrated that tacrolimus (TAC) or calcineurin inhibitors (such as cyclosporine [CSA]) can induce remission in most patients with IMN, the associated renal toxicity and high cost burden are major concerns.^[8,9]^ Therefore, it is necessary to explore other therapeutic strategies for treating IMN.

Tripterygium wilfordii Hook F (TwHF) is a member of the Celastraceae family of perennial vine-like plants. Tripterygium wilfordii multiglycosides is a preparation that is extracted and purified from the root xylem of TwHF and is sold as tablets. It is widely in-depth research in China, and is proved to have anti-inflammatory and immunosuppressive effect.^[10,11]^ It has been extensively used in China for the treatment of autoimmune diseases, such as rheumatoid arthritis,^[12–14]^ systemic lupus erythematosus (SLE),^[15]^ and nephrotic syndrome.^[16–20]^ We will perform a systematic review and meta-analysis to assess the strength of the current evidence to support the efficacy and safety of tripterygium wilfordii multiglycosides combined with prednisone to treat IMN, which might be a complementary therapy for IMN.

## 2 Methods

The review protocol has been registered with the International Prospective Register of Systematic Reviews (PROSPERO registration No.CRD42018118179; available online: http://www.crd.york.ac.uk/PROSPERO/myprospero.php). This article will be written following the Preferred Reporting Items for Systematic Reviews and meta-Analyses (PRISMA) reporting guidelines.^[21]^

### 2.1 Inclusion and exclusion criteria

Inclusion criteria: the study was a randomized controlled trial (RCT); the study examined IMN participants who received tripterygium wilfordii multiglycosides combined with prednisone; the study included participants irrespective of sex, age, or ethnicity and IMN was diagnosed by clearly defined or internationally recognized criteria.

Exclusion criteria: studies describing interventions combined with other TCM therapies such as Chinese herbal medicine, acupuncture, acupoint injection, or herbal extracts; studies that were non-randomized controlled trials and quasi-randomized controlled trials.

Outcomes: The primary outcomes are the complete remission rate (CR), remission rate (RR), and adverse events. CR refers to a decrease in the 24-hour urinary protein excretion (24h-UTP) to ≤0.3 g, whereas PR refers to a reduction in the urinary protein level to 0.3 to 3.5 g/d along with a 50% reduction from its peak values. RR = CR + PR. The secondary outcomes are 24h-UTP, ALB, TG.

### 2.2 Search strategy

The following 7 electronic databases will be searched to identify eligible trials published from inception to December 31, 2019: Embase, PubMed, Cochrane Database of Systematic Reviews, Web of Science, Chinese National Knowledge Infrastructure, Chinese Scientific Journal Database (VIP), and the Wanfang database. (Glomerulonephritides, Membranous [MeSH Terms]) OR Membranous Glomerulonephritides) OR Membranous Glomerulonephritis) OR Nephropathy, Membranous) OR Membranous Glomerulopathy) OR Glomerulopathy, Membranous) OR Membranous Nephropathy) OR Extramembranous Glomerulopathy) OR Glomerulopathy, Extramembranous) OR Membranous Glomerulonephropathy) OR Glomerulonephropathy, Membranous) OR Idiopathic Membranous Glomerulonephritis) OR Glomerulonephritides, Idiopathic Membranous) OR Glomerulonephritis, Idiopathic Membranous) OR Idiopathic Membranous Glomerulonephritides) OR Membranous Glomerulonephritides, Idiopathic) OR Membranous Glomerulonephritis, Idiopathic) OR Idiopathic Membranous Nephropathy) OR Membranous Nephropathy, Idiopathic) OR Nephropathy, Idiopathic Membranous) OR Heymann Nephritis) OR Nephritis, Heymann)) AND (Tripterygiums [MeSH Terms]) OR Tripterygium hypoglaucum) OR Tripterygium hypoglaucums) OR hypoglaucums, Tripterygium) OR Tripterygium wilfordii) OR Tripterygium wilfordius) OR wilfordius, Tripterygium) OR Leigong Teng) OR Leigong Tengs) OR Teng, Leigong) OR Tengs, Leigong) OR Thundergod Vine) OR Thundergod Vines) OR Vine, Thundergod) OR Vines, Thundergod) OR Tripterysium Glycosides) for English databases: (Leigongtengduogan) OR (Leigongtengduoganpian) AND (Moxingshenbing) OR (Moshen) OR (Shenbingzonghezheng). Two reviewers (JYX and ZJY) will independently screen the titles and abstracts for eligibility and examine the full text of the articles. Any discrepancies are resolved by consensus or after consulting a third party (ZQ).

### 2.3 Data extraction

**Figure 1:**
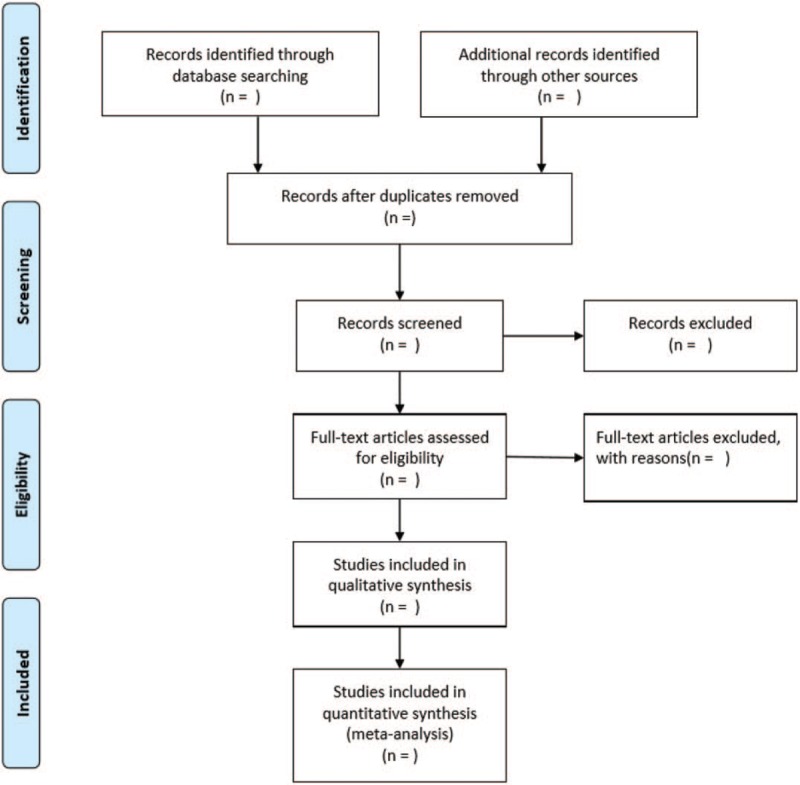
Flow of literature screening.

Two reviewers (JYX and WYX) will independently extract data using an extraction sheet. The extracted data includes general trial characteristics (authors, year); baseline patient and disease data (sample size, age, sex, disease duration); interventions and outcomes (treatment duration, outcome measures, adverse events). The entire process of study selection is performed in the PRISMA flow diagram (Fig. 1).

### 2.4 Quality assessment

Two reviewers (JYX and XX) will independently assess the methodological quality of the RCTs using the Cochrane Collaboration Risk of Bias tool. The risk of bias is assessed according to the Cochrane Handbook, which consists of 6 items: random sequence generation (i.e., selection bias), allocation concealment (i.e., selection bias), blinding of participants and personnel (i.e., performance bias), blinding of outcomes assessment (i.e., detection bias), incomplete outcomes data (i.e., attrition bias), selective reporting (i.e., reporting bias), and other biases. Discrepancies in this interpretation are resolved by consensus or after discussion with a third party (ZQ).

### 2.5 Measures of treatment effect

To summarize the effects of acupuncture treatment for each study, relative risk will be used when the result is dichotomous data. In cases with continuous data, the mean differences (MD) or the standard mean differences will be used. The effect sizes will be displayed using 95% confidence intervals.

### 2.6 Dealing with missing data

If data are missing or insufficient, we will contact the author by email or telephone to obtain the necessary information. If we fail to recover sufficient data, the data will be discarded. We will conduct our analysis based on available data, and the potential impact of missing data will be discussed.

### 2.7 Assessment of the quality of the evidence

The Grading of Recommendations Assessment, Development, and Evaluation (GRADE) method will be used to assess the quality of the evidence for each outcome. According to GRADE, the outcomes of an intervention are categorized into 4 levels of evidence quality: +very low, ++low, +++moderate, and ++++high. In GRADE, the confidence assessment addresses the risk of bias (in individual studies), inconsistency (heterogeneity in estimates of an effect across studies), indirectness (related to the question or due to intransitivity), imprecision, and publication bias. RCTs start as high quality evidence, whereas those from observational studies start as low quality evidence. Defined criteria are applied to either decrease or increase the quality of evidence rating. The GRADE profiler (GRADEPRO) will be applied to create the summary of evidence table.

### 2.8 Summary measures and data synthesis

The data will be analyzed using Review Manager 5.3 software (Cochrane Collaboration, Oxford, UK). The results are presented as the risk ratios (RR) or MD with the 95% confidence interval (95% CI). *I*^2^ statistics are used to assess heterogeneity. A fixed-effects (FE) model is used if there is no significant heterogeneity in the data (*I*^2^ < 50%), and a random-effects (RE) model is used if significant heterogeneity is present (*I*^2^ > 50%). Publication bias is assessed using funnel plots. Egger tests and Begg tests^[22,23]^ are conducted using Rversion3.3.2 to determine whether the funnel plots are symmetrical.

### 2.9 Subgroup analysis

Subgroup analysis will be performed to assess the high heterogeneity of included studies. We will conduct subgroup analysis based on the data, such as disease duration, intervention time, and so on.

### 2.10 Sensitivity analysis

After the quality assessment of the included literature, if there are possible low-quality studies, sensitivity analysis will be required. We will observe fluctuation of termination by changing the genre of research (incorporating or excluding a particular study) and reanalysis of simulated missing data.

## 3 Ethics and dissemination

Ethical approvals and patient consent are not necessary because the meta-analysis is based on published research. We will submit our meta-analysis to a peer-reviewed journal for publication.

## 4 Discussion

IMN is one of the most common causes of nephrotic syndrome. If no treatment is administered, IMN may lead to end-stage renal disease within 5 to 15 years. According to the KDIGO guidelines, the initial treatment regimen for IMN is oral or intravenous glucocorticoid and alkylating agents (alternate monthly), or alternative treatment with calcineurin inhibitor (CNI), and there are also experimental evidence that rituximab can also treat membranous nephropathy.^[24,25]^ However, the former has heavy adverse reactions^[8,9]^ while the latter is more expensive. In China, tripterygium wilfordii multiglycosides are widely used in autoimmune diseases and nephrotic syndrome, with few side effects and low price—1/6 of the price of CSA and 1/9 of the price of FK506. Therefore, tripterygium wilfordii multiglycosides are increasingly widely used in patients with IMN in China. However, few reviews have evaluated their effectiveness systematically. This review aims to objectively evaluate the effectiveness and safety of tripterygium wilfordii multiglycosides combined with prednisone in the treatment of idiopathic membranous nephropathy based on evidence-based medicine. Due to the particularity of medicine (TCM medicine), it is expected that the RCTs retrieved in this review are mostly conducted in China, and the differences in specific treatment regimens and methodological quality in each trial can lead to significant heterogeneity.

## Author contributions

JYX and ZQ designed the study. JYX and ZJY developed the search strategy. JYX and WYX wrote the manuscript. All authors provided critical revisions of the protocol and approved the final manuscript.
